# Investigation of Different Sparsity Transforms for the PICCS Algorithm in Small-Animal Respiratory Gated CT

**DOI:** 10.1371/journal.pone.0120140

**Published:** 2015-04-02

**Authors:** Juan F. P. J. Abascal, Monica Abella, Alejandro Sisniega, Juan Jose Vaquero, Manuel Desco

**Affiliations:** 1 Departamento de Bioingeniería e Ingeniería Aeroespacial, Universidad Carlos III de Madrid, Madrid, Spain; 2 Instituto de Investigación Sanitaria Gregorio Marañón (IiSGM), Madrid, Spain; 3 Centro de Investigación en Red de Salud Mental (CIBERSAM), Madrid, Spain; University of Nebraska Medical Center, UNITED STATES

## Abstract

Respiratory gating helps to overcome the problem of breathing motion in cardiothoracic small-animal imaging by acquiring multiple images for each projection angle and then assigning projections to different phases. When this approach is used with a dose similar to that of a static acquisition, a low number of noisy projections are available for the reconstruction of each respiratory phase, thus leading to streak artifacts in the reconstructed images. This problem can be alleviated using a prior image constrained compressed sensing (PICCS) algorithm, which enables accurate reconstruction of highly undersampled data when a prior image is available. We compared variants of the PICCS algorithm with different transforms in the prior penalty function: gradient, unitary, and wavelet transform. In all cases the problem was solved using the Split Bregman approach, which is efficient for convex constrained optimization. The algorithms were evaluated using simulations generated from data previously acquired on a micro-CT scanner following a high-dose protocol (four times the dose of a standard static protocol). The resulting data were used to simulate scenarios with different dose levels and numbers of projections. All compressed sensing methods performed very similarly in terms of noise, spatiotemporal resolution, and streak reduction, and filtered back-projection was greatly improved. Nevertheless, the wavelet domain was found to be less prone to patchy cartoon-like artifacts than the commonly used gradient domain.

## Introduction

**Fig 1:**
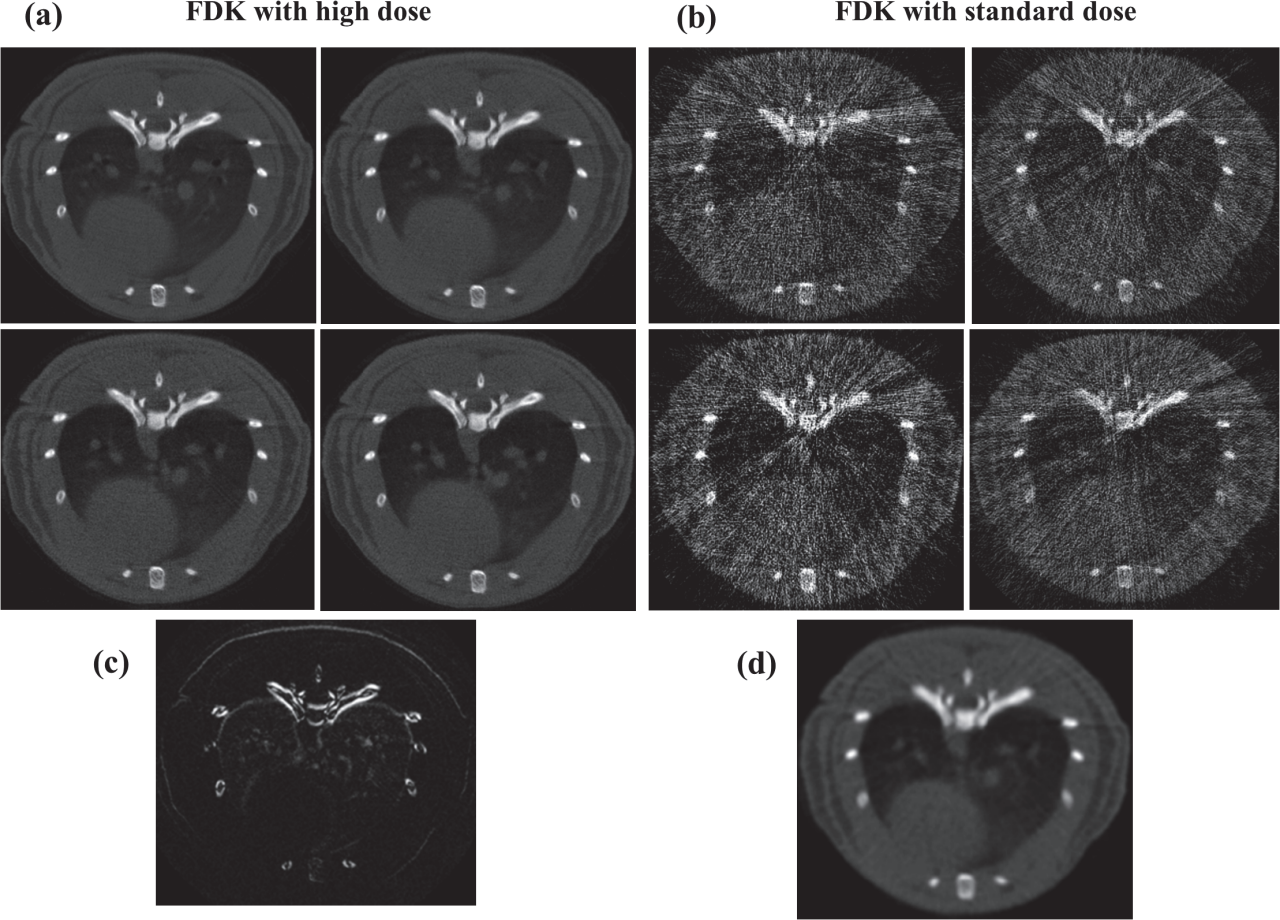
Comparison of static imaging and reference high-dose protocols. FDK reconstructions of gated data obtained with the reference high-dose protocol (A) and with the static imaging protocol (B) comprising 32 and 8 frames per projection angle, respectively. C: Absolute image difference between two high-dose respiratory phases. D: Prior image obtained from the addition of the four low-dose respiratory phases and processed with a Gaussian filter to reduce noise.

Respiratory gating helps to overcome the problem of breathing motion in cardiothoracic small-animal imaging. CT imaging is the gold standard in several lung diseases, such as tuberculosis. Blurring caused by breathing motion can hinder quantification in imaging studies, which are useful for assessing the degree of infection based on the density and extension of the lesions. One option for improving image quality is to correct movement blurring using retrospective gating. If we generate complete data sets for a number of respiratory phases by acquiring multiple images for each projection angle [1], the radiation dose delivered to the subject increases proportionally to the number of respiratory phases. Fig 1(A) shows an example of 4 respiratory phases obtained with 32 images per projection angle using a high-dose protocol. If we use only 8 frames per projection angle (corresponding to a dose similar to that of a static imaging protocol), few noisy and irregularly distributed projections are available for the reconstruction of each respiratory phase, thus leading to streak artifacts in the FBP-reconstructed images (Fig 1(B)). In previous approaches [2, 3], this problem was solved within an analytical framework using a variation of the McKinnon-Bates method [4], which is based on correction of an initial estimate obtained from the whole data set (combining all respiratory phases) with the undersampled data from each respiratory phase. Although this approach reduces the presence of noise and artifacts, correcting the artifacts present in the initial estimate remains challenging [2].

Correction of respiratory motion has been addressed for image-guided radiotherapy using non-rigid image registration based on a motion model [5, 6]. In order to reduce the effect of the streak artifacts, the registration was restricted to a volume of interest, defined by a boundary set 2 mm outside the body of the subject. Finally, the registered images were combined and residual streak artifacts further reduced using principal component analysis. The drawback of this approach is that it requires good image quality to guide non-rigid registration.

From a different perspective, in the compressed sensing (CS) framework, an image can be accurately reconstructed from few projections using convex optimization, provided that the image is sparse in a transformed domain [7–11]. The most commonly used transformed domain is the gradient that leads to total variation (TV) [12–14], which efficiently removes noise and artifacts caused by undersampling, but leads to patchy images for high undersampling factors [15].

A combination of both strategies, i.e., prior image and sparsity, is found in the so-called prior image constrained compressed sensing (PICCS) algorithm [16–18], which enables accurate reconstruction of highly undersampled data. PICCS combines TV, which removes noise, with a prior image that helps to maintain a natural image texture. This prior image is usually obtained as the average of all respiratory phases, similar to the procedure followed in the McKinnon-Bates method [4]. PICCS has been applied to contrast cardiac CT data [16–18] and respiratory gated phantom data [19]. It has also been applied to characterize breathing motion and requires a lower acquisition time than filtered back projection with McKinnon-Bates correction [20]. Several works have proposed variations of PICCS: an adaptive PICCS for longitudinal CT studies [21], an extension to include a log-likelihood–based fidelity term [22], and a nonconvex approach [23].

The PICCS algorithm is a constrained optimization based on L1-penalty functions that can be solved using classic constrained optimization methods. However, since these methods can be computationally expensive, most algorithms solve the constrained TV problem using methods that alternate steepest descent for minimization of an unconstrained version of TV with iterative methods such as the simultaneous algebraic reconstruction technique (SART), which imposes fidelity on the acquired data. Algorithms such as adaptive steepest descent projection onto convex sets (ASD-POCS) are based on this approach [24]. Nevertheless, solving an unconstrained approximated version of the constrained problem requires optimal selection of the regularization parameter and estimation of the step size. The Split Bregman algorithm was applied to MRI [25, 26] and proved to be optimal and computationally efficient for the solution of constrained problems with L1-penalty functions. In addition, this approximation facilitates the enforcement of constraints and it circumvents the requirement of an optimal selection of the regularization parameter. Similar alternating methods were also applied to CT [27].

With regard to sparsity transforms, the preferred choice for CT is the gradient domain. Although other transforms may be sparser, depending on the application, few studies have actually used a different choice, and even fewer have offered a comparison. In [28], the authors propose the shearlet transform for static CT, and in [29], wavelet frames were tested on phantom data. In the case of the PICCS method, to our knowledge, no studies have evaluated different sparsity transforms.

In this study, we compare three versions of the PICCS algorithm using different transforms in the prior penalty function term (unitary, gradient, and wavelet). In all three cases, the problem was solved using the Split Bregman formulation. In addition, positive and support constraints were added to the standard PICCS method. The evaluation was performed on small-animal CT data in terms of contrast in bone and lung tissue, mean square error (MSE) with respect to the target, image noise, contrast-to-noise ratio (CNR), degree of compensation of the respiratory movement, and image texture quality. We also analyzed the performance of the algorithm for different weights of the prior penalty term and studied different settings of X-ray flux (related to delivered dose) and number of projections. Preliminary results were presented earlier for a fixed flux and number of projections [30].

## Methods

### Image reconstruction

#### PICCS

The PICCS method can be used to reduce streak artifacts and noise when reconstructing highly undersampled gated-CT data. With *u*_*i*_ as the *i-*th phase image, PICCS assumes that *u*_*i*_ is sparse in a transformed domain *T*_1_ and that there must be a prior image *u*_*p*_ to ensure that *u*_*i*_–*u*_*p*_ is sparse in a transformed domain *T*_2_. If *f*_*i*_ represents the data corresponding to the *i-*th phase image and *F* is the forward operator, PICCS is the convex constrained optimization problem

$$\underset{u_{i}}{\text{min}}(1-\alpha )\|T_{1}u_{i}{\|}_{1}+\;\alpha \|T_{2}(u_{i}-u_{p}){\|}_{1}\text{ such that }\|Fu_{i}-f_{i}{\|}^{2}\leq \sigma^{2},\text{ i}=\text{1,}\ldots ,I$$ (1)

where *I* is the total number of respiratory phase bins, σ accounts for noise in the data and α weights the prior penalty function. The common choice for *T*_1_ is the spatial discrete gradient that leads to TV, ||∇*u*_*i*_||_1_, which filters out noise while preserving edges in the image. TV is also a common choice for *T*_2_.

We remark that, for α = 0, the problem in Equation (1) corresponds to the minimization of TV subject to a data constraint. TV assumes that the image is piecewise smooth and has been shown to yield ‘cartoonish’ images for a low number of projections [15]. When 0 < *α* ≤ 1, the addition of the prior image prevents the excessive smoothing produced by TV and helps to maintain the texture of the prior image.

#### PICCS with positivity and support constraints solved using the Split Bregman formulation

We extended the PICCS method by adding a positivity constraint [31, 32] and a support constraint that restrict the reconstruction to a circular field of view, Ω, defined by the Radon transform. Thus, the reconstruction problem in Equation (1) becomes

$$\underset{u_{i}}{\text{min}}(1-\alpha ){\left\|\nabla u_{i}\right\|}_{1}+\;\alpha {\left\|T_{2}(u_{i}-u_{p})\right\|}_{1}\text{ such that }{\left\|Fu_{i}-f_{i}\right\|}^{2}\leq \sigma^{2},\;u_{i}\geq 0,\;u_{i}\in \Omega$$ (2)

where we use isotropic TV, ${\left\|\nabla u_{i}\right\|}_{1}=\sqrt{{\left(\nabla_{x}u_{i}\right)}^{2}+{\left(\nabla_{y}u_{i}\right)}^{2}}$.

To solve the problem in Equation (2), we use the Split Bregman formulation, which efficiently handles L1-based constrained problems [25, 33]. The Split Bregman formulation makes it possible to split L1-norm terms and L2-norm terms in such a way that they can both be solved analytically in two separate steps. The part including the L2-norm functionals results in a linear system that can be solved using linear iterative methods, and the part with L1-norm functionals is solved using shrinkage formulas, as shown below.

To allow for splitting, we include new variables, *d*_*xi*_, *d*_*yi*_, *w*_*i*_ and *v*_*i*_, and formulate a new problem that is equivalent to Equation (2)

$$\begin{array}{c} \underset{u_{i},d_{xi},d_{yi},v_{i},w_{i}}{\min}(1-\alpha ){\left\|\left(d_{xi},d_{yi}\right)\right\|}_{1}+\;\alpha {\left\|w_{i}\right\|}_{1}\text{ such that}\\ \;{\left\|Fu_{i}-f_{i}\right\|}^{2}\leq \sigma^{2},\;v_{i}\geq 0,\;v_{i}\in \Omega ,\;d_{xi}=\nabla_{x}u_{i},\;d_{yi}=\nabla_{y}u_{i},\;w_{i}=T_{2}(u_{i}-u_{p}),\;v_{i}=u_{i} \end{array}$$ (3)

Equation (3) is easily managed using an equivalent unconstrained optimization approach with constraints imposed by adding a Bregman iteration *b*_*i*_ for each constraint. That is,

$$\begin{array}{l} \underset{u_{i},d_{xi},d_{yi},v_{i},w_{i}}{\min}\text{ (1-}\alpha ){\left\|\left(d_{xi},d_{yi}\right)\right\|}_{1}+\;\alpha {\left\|w_{i}\right\|}_{1}\;+\Phi \left({\text{v}}_{\text{i}}\geq 0,v_{i}\in \Omega \right)+\frac{\mu }{2}{\left\|Fu_{i}-f_{i}^{k}\right\|}_{2}^{2}+\frac{\lambda }{2}{\left\|d_{xi}-\nabla_{x}u_{i}-b_{xi}^{k}\right\|}_{2}^{2}+\\ \frac{\lambda }{2}{\left\|d_{yi}-\nabla_{y}u_{i}-b_{yi}^{k}\right\|}_{2}^{2}+\frac{\lambda }{2}{\left\|w_{i}-T_{2}(u_{i}-u_{p})-b_{{}_{wi}}^{k}\right\|}_{2}^{2}+\frac{\gamma }{2}{\left\|v_{i}-u_{i}-b_{vi}^{k}\right\|}_{2}^{2} \end{array}$$ (4)

where Φ(v_i_ ≥ 0, *v*_*i*_ ∊ Ω) represents the non-negativity and support constraints, *k* is the iteration number and the Bregman iterations are updated as

$$\begin{array}{c} b_{xi}{}^{k+1}=b_{xi}{}^{k}+\nabla_{x}u_{i}^{k+1}-d_{xi}{}^{k+1}\\ b_{yi}{}^{k+1}=b_{yi}{}^{k}+\nabla_{y}u_{i}^{k+1}-d_{yi}{}^{k+1}\\ \begin{array}{l} b_{wi}{}^{k+1}=b_{wi}{}^{k}+T_{2}(u_{i}^{k+1}-u_{p})-w_{i}^{k+1}\\ b_{vi}{}^{k+1}=b_{vi}{}^{k}+u_{i}^{k+1}-v_{i}^{k+1}\\ f_{i}^{k+1}=f_{i}^{k}+f_{i}-Fu_{i}^{k+1} \end{array} \end{array}$$ (5)

The Bregman iteration imposes the constraints iteratively by adding the error back into the constraints. Thus, introducing the Bregman iteration into the unconstrained formulation [Equation (4)] forces its solution to converge to the solution of the constrained problem [Equation (2)] for sufficiently small values of the parameters μ, λ, and γ. The data constraint in Equation (5) leads to a sequence of solutions for which both the solution error norm and the data fidelity term decrease monotonically. This formulation is more robust than equivalent approximated unconstrained problems or continuation methods that impose the constraint iteratively by slowly increasing the regularization parameters [25].

Note that, as *u*_*i*_ and the auxiliary variables are independent of each other, Equation (4) can now be split into several equations (one for each variable) that are solved sequentially, as follows:

$$\begin{array}{l} u_{i}{}^{k+1}=\underset{u_{\text{i}}}{\min}\;\frac{\mu }{2}{\left\|Fu_{i}-f_{i}^{k}\right\|}_{2}^{2}+\frac{\lambda }{2}{\left\|d_{xi}{}^{k}-D_{x}u_{i}-b_{xi}^{k}\right\|}_{2}^{2}+\frac{\lambda }{2}{\left\|d_{yi}{}^{k}-D_{y}u_{i}-b_{yi}^{k}\right\|}_{2}^{2}+\frac{\lambda }{2}{\left\|w_{i}{}^{k}-T_{2}(u_{i}-u_{p})-b_{{}_{wi}}^{k}\right\|}_{2}^{2}+\\ \;+\frac{\gamma }{2}\;{\left\|v_{i}{}^{k}-u_{i}-b_{vi}^{k}\right\|}_{2}^{2}\\ d_{xi}{}^{k+1},d_{yi}{}^{k+1}\underset{d_{xi},d_{yi}}{=\min}\text{ (1-}\alpha ){\left\|\left(d_{xi},d_{yi}\right)\right\|}_{1}+\frac{\lambda }{2}{\left\|d_{xi}-D_{x}u_{i}{}^{k+1}-b_{xi}^{k}\right\|}_{2}^{2}+\frac{\lambda }{2}{\left\|d_{yi}-D_{y}u_{i}{}^{k+1}-b_{yi}^{k}\right\|}_{2}^{2}\\ w_{i}{}^{k+1}=\underset{w_{i}}{\min}\;\alpha {\left\|w_{i}\right\|}_{1}\;+\frac{\lambda }{2}{\left\|w_{i}-T_{2}(u_{i}^{}{}^{k+1}-u_{p})-b_{{}_{wi}}^{k}\right\|}_{2}^{2}\\ v_{i}{}^{k+1}=\underset{v_{i}}{\min}\;\frac{\gamma }{2}\;{{\left\|v_{i}-u_{i}{}^{k+1}-b_{vi}^{k}\right\|}_{2}^{2}|}_{{\text{v}}_{\text{i}}\geq {\text{0,v}}_{\text{i}}\in \Omega }\; \end{array}$$ (6)

Since solution of *u*_*i*_ only involves L2-norm functionals, it can be determined exactly by differentiating the cost function and equating it to zero. The result is a linear system that corresponds to a Gauss-Newton step

$$\begin{array}{l} Ku_{i}^{k+1}=r_{i}^{k}\\ K=\mu F^{T}F+\lambda D_{x}{}^{T}D_{x}+\lambda D_{y}{}^{T}D_{y}+\lambda T_{2}{}^{T}T_{2}+\gamma I\\ r_{i}^{k}=\mu F^{T}f_{i}^{k}+\lambda D_{x}{}^{T}\left(d_{xi}^{k}-b_{xi}^{k}\right)+\lambda D_{y}{}^{T}\left(d_{yi}^{k}-b_{yi}^{k}\right)+\lambda T_{2}{}^{T}\left(w_{i}^{k}+T_{2}u_{p}-b_{wi}^{k}\right)+\gamma \left(v_{i}^{k}-b_{vi}^{k}\right) \end{array}$$ (7)

Note that Equation (7) is an analytical function, so an estimation of the step-size is not required. This linear system constitutes a very large-scale problem, where K = NxN, being N the number of pixels, yet it can be solved efficiently using a Krylov solver that involves only matrix-vector multiplications:

$$\mu F^{T}(Fu_{i})+\lambda D_{x}{}^{T}(D_{x}u_{i})+\lambda D_{y}{}^{T}(D_{y}u_{i})+\lambda T_{2}{}^{T}(T_{2}u_{i})+\gamma u_{i}=r_{i}$$ (8)

Here, we used the biconjugate gradient stabilized method with a threshold δ in the range of 10^–2^ to 10^–4^, where δ = 10^–2^ accelerated convergence (four to six iterations).

*d*_*xi*_, *d*_*yi*_, *v*_*i*_ and *w*_*i*_ are solved analytically using shrinkage formulas, which are thresholding operations [Goldstein 2009, Wang 2008]

$$\begin{array}{c} d_{xi}{}^{k+1},d_{yi}{}^{k+1}=\text{m}ax\left(s_{i}{}^{k}\text{-(1-}\alpha \text{)/}\lambda ,0\right)\frac{\left|D_{j}u_{i}{}^{k+1}+b_{ij}^{k}\right|}{s_{i}{}^{k}},\;s_{i}{}^{k}=\sqrt{{\left|D_{x}u_{i}{}^{k+1}+b_{ix}^{k}\right|}^{2}+{\left|D_{y}u^{k+1}+b_{iy}^{k}\right|}^{2}},\;j=x,y\\ w_{i}{}^{k+1}=\text{shrink(}T_{2}(u_{i}{}^{k+1}-u_{p})+b_{wi}^{k}\text{,}\alpha \text{/}\lambda \text{)}=\text{max}\left(\left|T_{2}(u_{i}{}^{k+1}-u_{p})+b_{wi}^{k}\right|\text{-}\alpha \text{/}\lambda ,0\right)\text{ sign}\left(T_{2}(u_{i}{}^{k+1}-u_{p})+b_{{}_{wi}}^{k}\right)\\ v_{i}{}^{k+1}=\text{m}ax(u_{i}{}^{k+1}+b_{vi}^{k}{\text{,0), v}}_{\text{i}}{\text{|}}_{{\text{v}}_{\text{i}}\notin \Omega }=0. \end{array}$$ (9)

### Evaluation

#### Test data: Simulation of different scenarios

Algorithms were evaluated using simulations generated from data acquired from a 10-week old adult female Wistar rat weighing 300 g, anaesthetized with isoflurane. Animals were handled according to the European Communities Council Directive (86/609/EEC) and with the approval of the Animal Experimentation Ethics Committee of Hospital General Universitario Gregorio Marañón (ES 280790000087).

The CT subsystem used for data acquisition was ARGUS PET/CT (SEDECAL), a cone-beam micro-CT scanner based on a flat panel detector [34]. The acquisition comprised 360 views of 512×512 pixels (0.2 mm^2^ pixel size) covering 360 degrees with 32 images per projection angle, at a source voltage of 45 kV.

Gated CT data were acquired using a high-dose protocol (four times the dose of a static imaging protocol) [1]. These high-dose projection data were arranged into four phases using software-based retrospective gating [1] and reconstructed with an FDK-based algorithm [35]. The resulting images were selected as our target.

These data were used to simulate scenarios with different X-ray flux levels and number of projections. Low-dose acquisitions were simulated by selecting a smaller field-of-view of 350x350 pixels (in order to reduce the computational cost), randomly taking 120 projections or less for each phase, and adding Poisson noise by modelling the measurements *f*_*i*_ as independently distributed Poisson random variables:

$$f_{i}∼Poisson\left\{{\bar{f}}_{i}\right\}\;i=1,\ldots ,M\text{ with }{\bar{f}}_{i}=I_{0}\;e^{-\int u\left(x,y,z\right)}$$ (10)

where *u*(*x*,*y*,*z*) is the high-dose reconstruction, *I*_0_ is the number of photons emitted by the x-ray source and M is the number of measured projections.

We simulated five different scenarios varying the number of photons emitted by the x-ray source (*I*_0_ = 4.5∙10^4^, *I*_0_/2, and *I*_0_/4 photons) and the number of projections per phase (120, 80 and 60 projections). *I*_0_ was chosen so as to obtain a noise figure for the prior image similar to that of the target (high-dose data). The number of projections per phase will depend on the time resolution of the respiratory cycle, i.e. the number of respiratory phases. Simulations were computed using the IRT code (J.A. Fessler, Image reconstruction toolbox (IRT), 2011, retrieved from <http://www.eecs.umich.edu/~fessler/code/index.html>).

Fig 1(A) shows four respiratory phases for the high-dose protocol and Fig 1(C) shows the image difference between two phases. Most differences between phases are within the lung area due to the respiratory movement and result in blurring of the prior image. Reconstruction of the low-dose respiratory phases with FDK led to images with noise and streak artifacts (Fig 1(B)). The prior was obtained by adding data from all phases and applying a Gaussian filter with σ = 5 to reduce noise (Fig 1(D)).

#### Comparison of methods and analysis of images

The low-dose data were reconstructed with three algorithms based on the PICCS approach—the unitary transform (L1-PICCS), the gradient transform (TV-PICCS), and the wavelet transform (WT-PICCS)—by varying transform *T*_2_. The three variations of PICCS based on Equation (2) were solved using the Split Bregman formulation [Equations (4–9)]. For the wavelet transform, we used the symmlet-8 base [36].

It is necessary to select the reconstruction parameters in Equation (6), namely, *k*, α, μ, λ, and γ. The iteration number *k* was chosen as the number of iterations that yielded the minimum mean-square error with respect to the reference high-dose image. The regularization parameter, μ, was selected following suggestions from previous studies [31, 33], which showed that for sufficiently small values of μ (μ ≤10 in our case), the problem converges to the same solution, albeit at a higher iteration number. The regularization parameter has the opposite effect. Low λ values (λ≤0.1) resulted in noisy images, as lower weight was given to the TV. For large λ values (λ≥1), the problem converges to similar results, although at a different iteration number. With regard to the regularization parameter γ, we found that low γ values (γ ≤1) were preferable because higher values impaired convergence. Given these considerations, we empirically selected μ = 10, λ = 1, and γ = 0.1.

Finally, to select the value of the prior weight, α, we analyzed the α effect by computing the two methods for α = 0.2, α = 0.5, and α = 0.8. In order to use the same range of parameters for all data sets, the algorithm normalizes the data item f as *f*/ǁ*f*/*n*ǁ, where *n* is the square root of the number of pixels in the image, following the suggestions from [Tom Goldstein. Split Bregman. Retrieved in 2009 from http://www.ece.rice.edu/~tag7/Tom_Goldstein/Split_Bregman.html].

**Fig 2:**
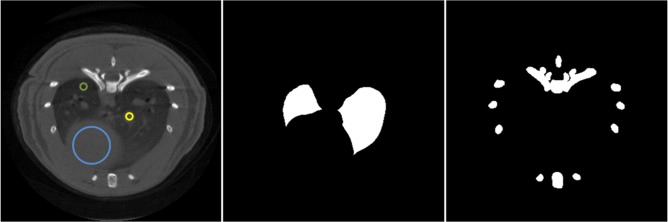
. Masks for quantitative analysis. Left: Mask used to measure contrast-to-noise ratio as the absolute difference between the yellow and green ROIs divided by the noise measured in the blue ROI. Middle: Mask to compute MSE in the lung area. Right: Masks to compute MSE in the bone area.

Images were compared in terms of several quantitative parameters: 1) Contrast in lung and bone areas measured as peak-to-valley (PV) on image profiles. 2) Noise, measured as the coefficient of variation in three different oval 312-pixel ROIs inside soft tissue. 3) Contrast-to-noise ratio, measured as the absolute difference between the value within a vessel and the value in a lung-tissue ROI divided by the noise (Fig 2 left). 4) Reconstruction error, assessed as mean-square error with respect to the target image (high-dose protocol) for both the lung area and bone, measured in the masks shown in Fig 2. 5) Movement compensation was assessed by drawing two profiles on moving areas, one across the vertebrae and another crossing a vessel inside the lung, and comparing them with the same profiles in the target image. In addition, image texture was evaluated by visual inspection.

## Results

### Evaluation of the influence of the parameter α

**Fig 3:**
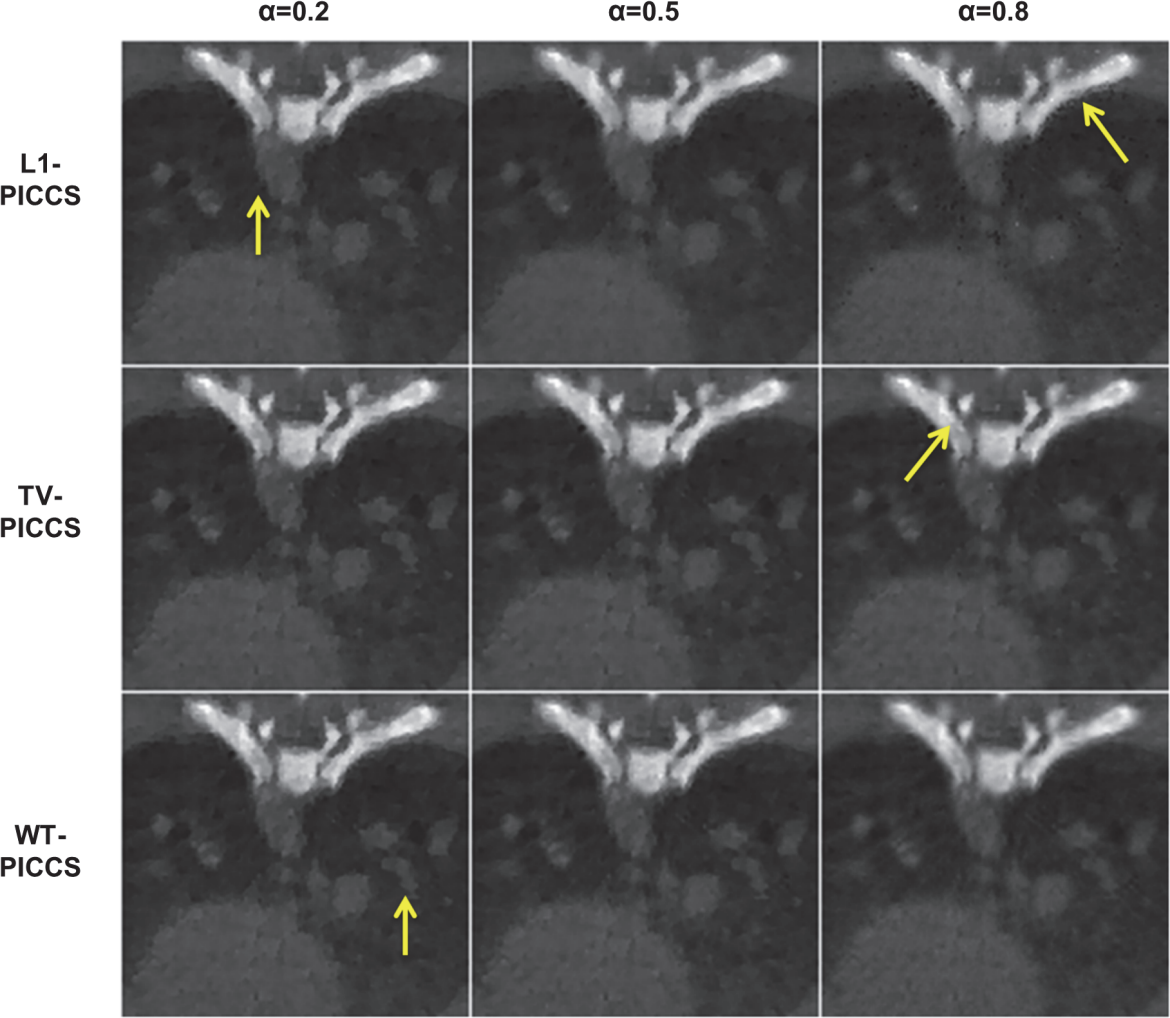
Zoomed-in images of the low-dose protocol data for respiratory phase one. Reconstructions were performed with L1-PICCS, TV-PICCS, and WT-PICCS for α equal to 0.2, 0.5, and 0.8, for 120 projections and X-ray flux corresponding to a number of photons *I*_0_ = 4.5∙10^4^. Arrows point at locations where differences in texture are more noticeable.

Fig 3 shows zoomed-in images of the best result for each CS method, for different values of the prior weight α. For small values of α, the prior term has a small influence and all CS methods converge to a solution similar to that provided by spatial TV, with a noticeable patchy pattern (α = 0.2, first column of Fig 3). Larger α values increase weight to the prior and leads to differences in the image texture depending on the sparsity transform used for this term. For α = 0.5 or 0.8, some differences in image texture are visible: L1-PICCS shows salt-and-pepper artifacts and TV-PICCS shows a patchy-like pattern (arrows in column 3 of Fig 3), while WT-PICCS shows a more natural texture.

From here onwards we choose α = 0.8 and discard L1-PICCS, which led to the largest artifacts.

### Analysis of the influence of X-ray flux and number of projections

**Fig 4:**
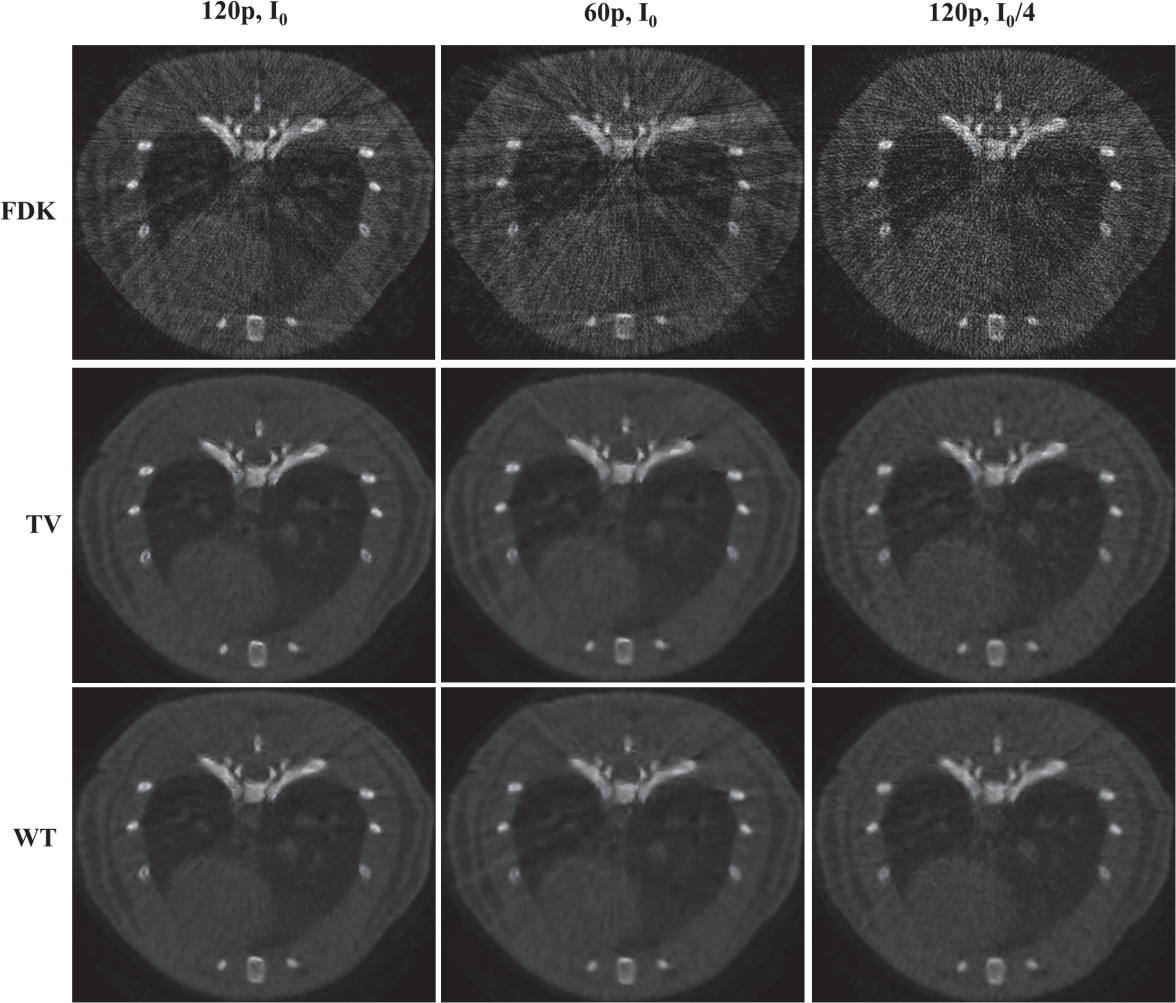
Images of several low-dose protocol data for respiratory phase one reconstructed with FDK, TV-PICCS, and WT-PICCS (α equal to 0.8). Each column represents a different scenario: 120 projections and flux corresponding to a maximum number of photons *I*_0_ (*I*_0_ = 4.5∙10^4^), 60 projections and number of photons *I*_0_, and 120 projections and number of photons *I*_0_/4 (from left to right).

Fig 4 shows the differences between FDK, TV-PICCS, and WT-PICCS reconstructions when decreasing the X-ray flux and the number of projections.

**Fig 5:**
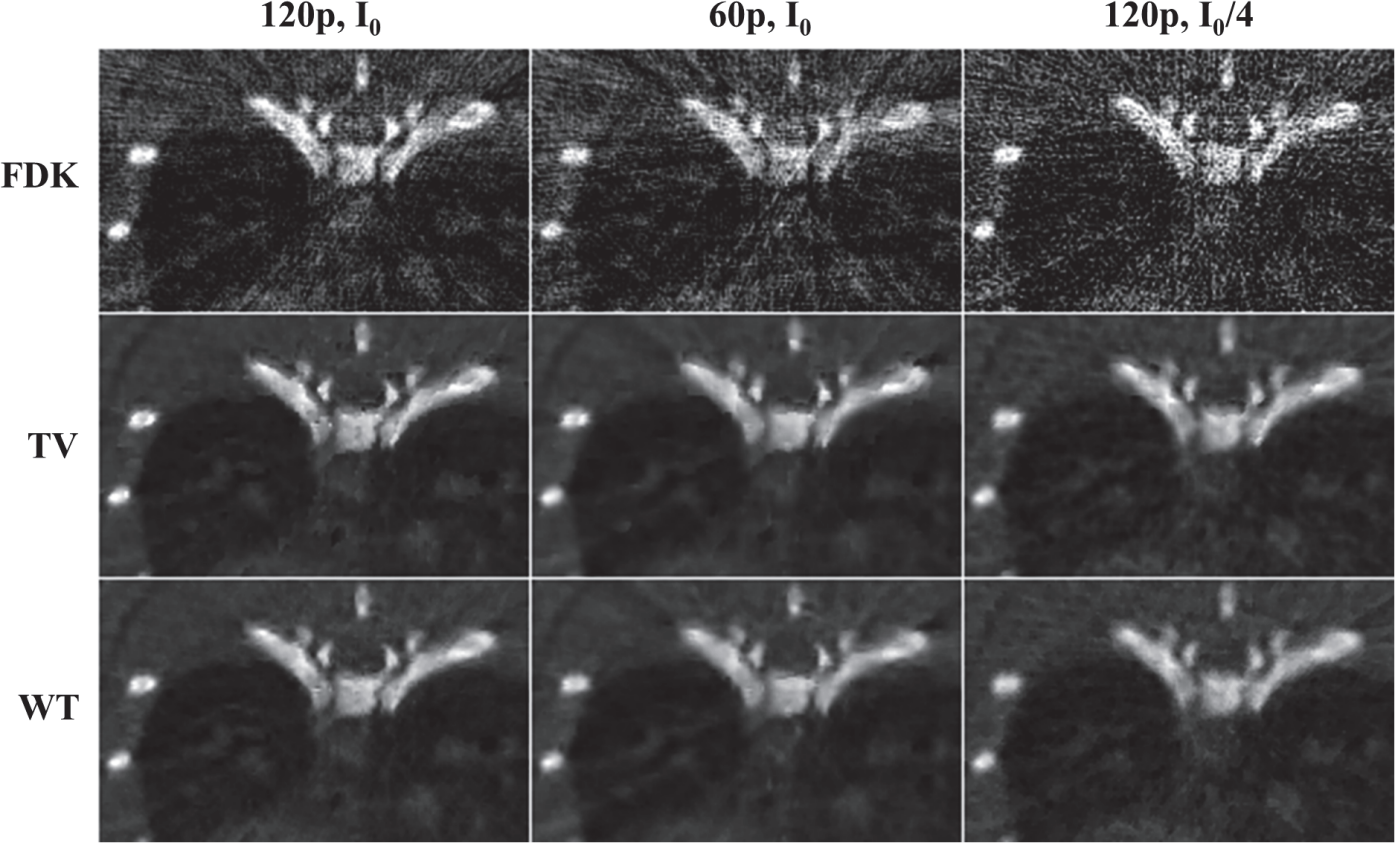
Zoomed-in images of low-dose protocol data for respiratory phase one, reconstructed with FDK, TV-PICCS, and WT-PICCS. Columns represent the different scenarios: 120 projections and dose corresponding to a maximum number of photons I_0_ (*I*_0_ = 4.5∙10^4^), 60 projections and number of photons *I*_0_, and 120 projections and number of photons I_0_/4 (from left to right). TV-PICCS and WT-PICCS were obtained with α = 0.8.

**Fig 6:**
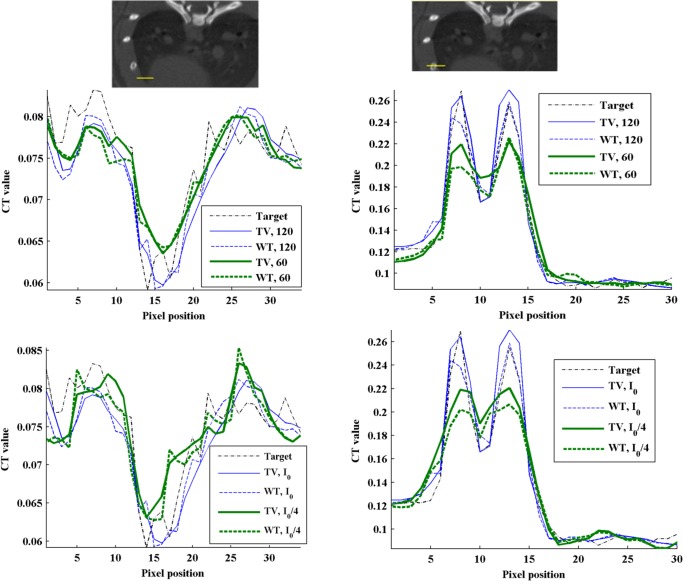
Influence of the number of projections and X-ray flux on image profiles. Top: Profile (yellow line) drawn over heart and lung areas (left figure) and profile drawn over a bone area (right figure), overimposed on the high-dose protocol image. Middle and bottom: Normalized profiles for reference high-dose FDK (target) and for the low-dose protocol reconstructed with TV-PICCS (TV) and WT-PICCS (WT) for different number of projections (middle) and different X-ray flux values (bottom).

Fig 5 shows zooms of Fig 4 to better depict differences between TV-PICCS and WT-PICCS when varying the source flux and number of projections. When decreasing the X-ray flux (third column in Fig 5), both algorithms converged in fewer iterations towards more blurred images (the loss of resolution can also be seen in the profile plotted in Fig 6). As the iteration number increases, the sparsity is imposed in the different domains. In the case of TV-PICCS, the algorithm leads to patchy artifacts for all noise levels tested, although with high noise the patchy artifact is less evident. WT- PICCS is more robust against noise level and maintains a more natural texture for all three scenarios. Varying the number of projections has a similar effect on texture: TV-PICCS presents patchy artifacts while WT-PICCS maintains a more natural texture. As the number of projections decreases, the missing data produce streaks that are not removed by any of the algorithms due to the coherent nature of this artifact, which cannot be removed by the TV term common to both algorithms.

Fig 6 illustrates the effect of the different number of projections and X-ray flux on an image profile drawn over lung and bone areas. Lowering either the number of projections or the X-ray flux reduces the recovered contrast in lung and bone. Decreasing the number of projections led to a 40% reduction in PV ratio in the lung profile with respect to the target, with no differences between the methods. For bone, decreasing the number of projections led to a 40% reduction in PV ratio using WT-PICCS and a 27% reduction using TV-PICCS.

**Fig 7:**
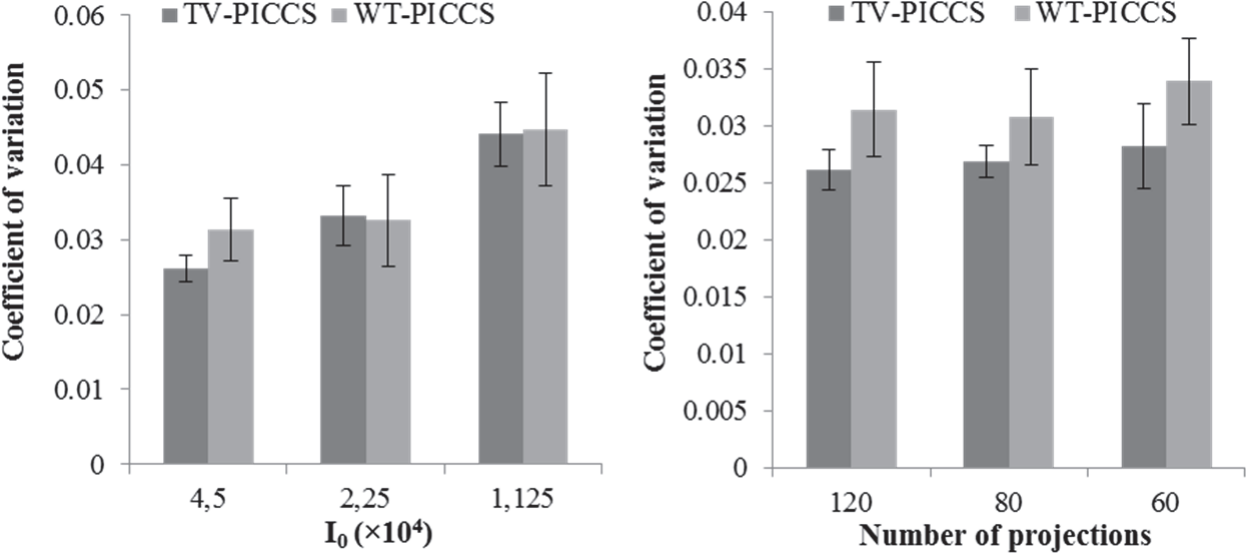
Coefficient of variation measured in images reconstructed with TV-PICCS and WT-PICCS for the different scenarios. Plots show mean and standard deviation of the coefficient of variation measured in three different 312-pixel ROI inside soft-tissues. Left panel shows different X-ray flux values for 120 projections; right panel represents different number of projections for a flux corresponding to a number of photons *I*_0_ = 4.5∙10^4^.

Fig 7 shows the image noise for all scenarios. Increasing the X-ray flux leads to less image noise for both TV-PICCS and WT-PICCS. Decreasing the number of projections does not have a significant influence on the image noise.

**Fig 8:**
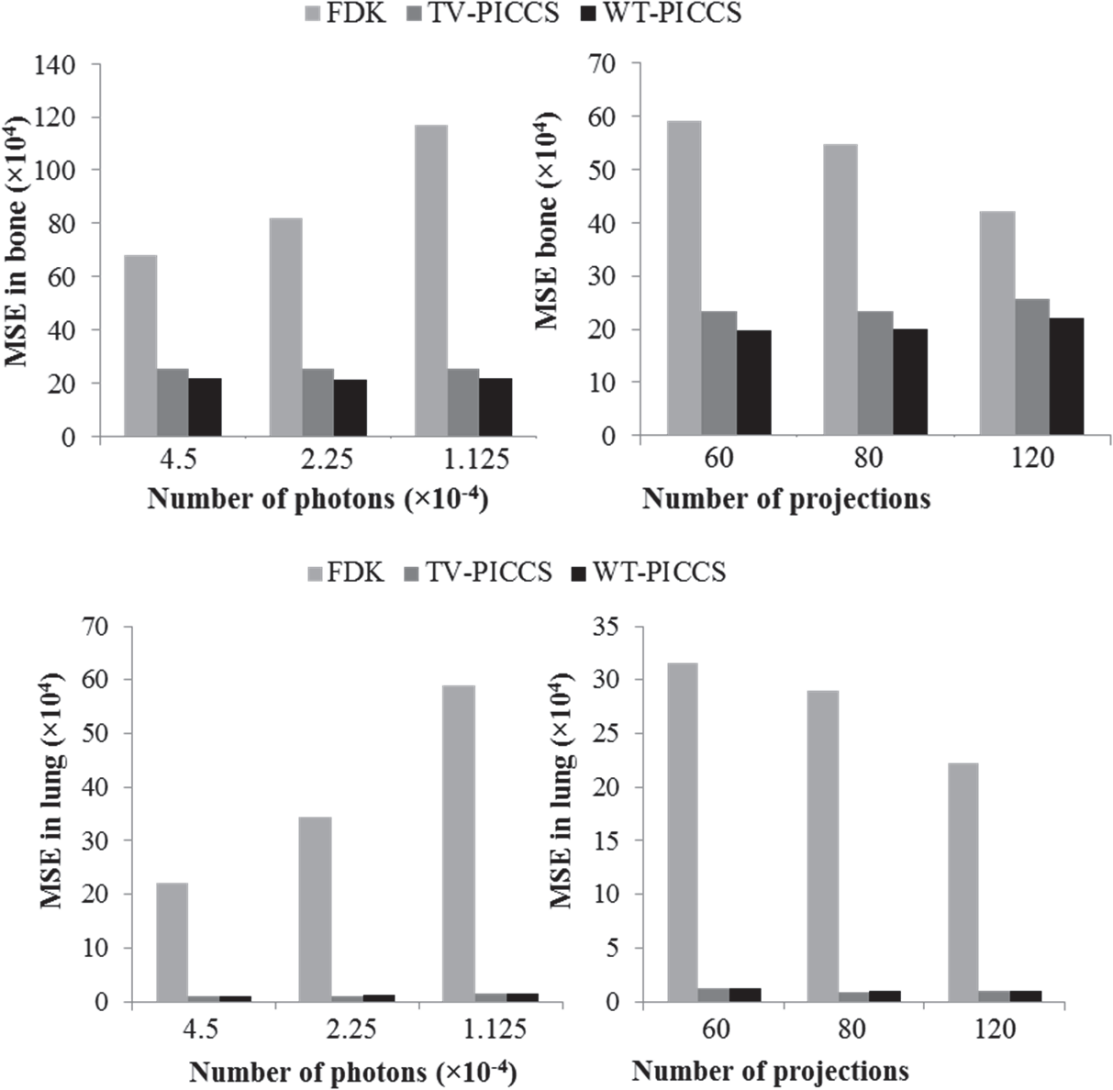
MSE with respect to the reference high-dose image for FDK, TV-PICCS and WT-PICCS for the different scenarios. Plots show MSE in bone tissue (top) and lung tissue (bottom) in the ROIs defined in Fig 2. Left panel shows different X-ray flux values for 120 projections; right panel represents different number of projections for an X-ray flux corresponding to a number of photons *I*_0_ = 4.5∙10^4^.

Fig 8 shows MSE for bone and soft tissue in different scenarios. For bone tissue, TV-PICCS and WT-PICCS showed a MSE reduction of 83% for the lowest the X-ray flux and of 67% for 60 projections, with respect to FDK reconstruction. There are no large differences between TV-PICCS and WT-PICCS, although WT-PICCS presented a slightly lower MSE. For lung tissue both MSE and PICCS showed an MSE sixty times lower than that of FDK.

**Fig 9:**
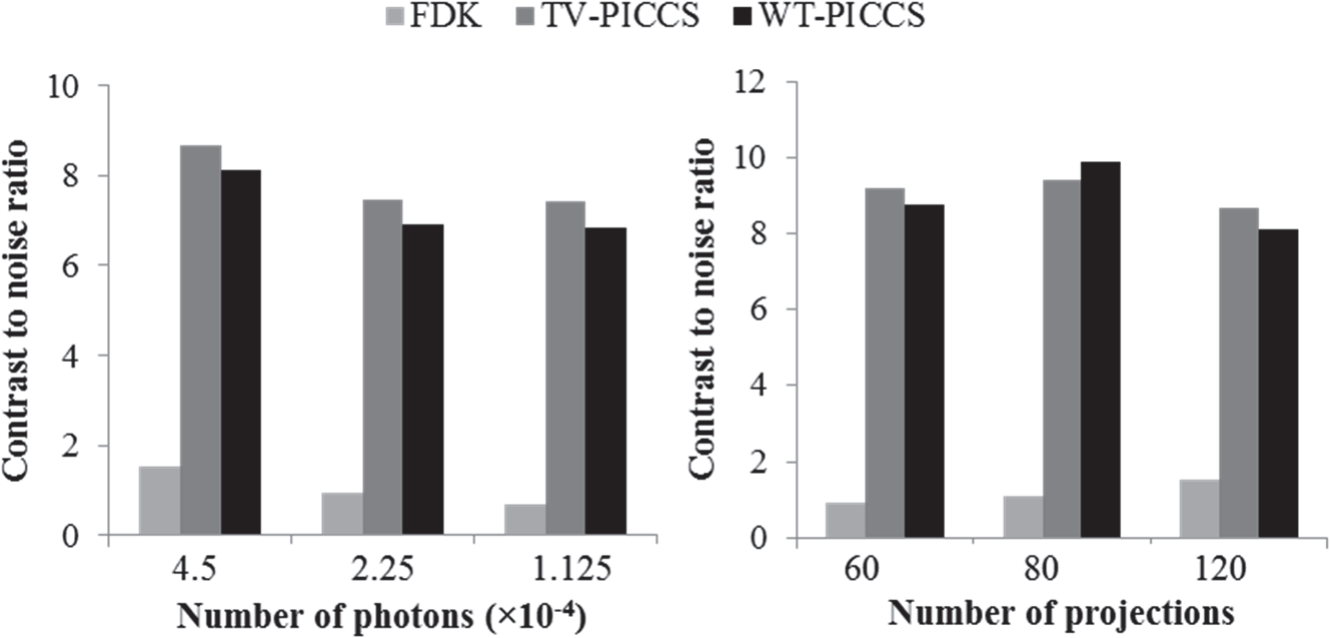
. Contrast-to-noise ratio measured in lung tissue in images reconstructed with FDK, TV-PICCS and WT-PICCS for the different scenarios. Left panel shows in the results for different X-ray flux values for 120 projections; right panel represents different number of projections for an X-ray flux corresponding to a number of photons *I*_0_ = 4.5∙10^4^.

Fig 9 shows the contrast-to-noise ratio of nodules in the lung. Both TV-PICCS and WT-PICCS lead to a ten-fold increase in CNR with respect to FDK, where TV-PICCS has slightly higher CNR than WT-PICCS.

### Analysis of the respiratory movement compensation

**Fig 10:**
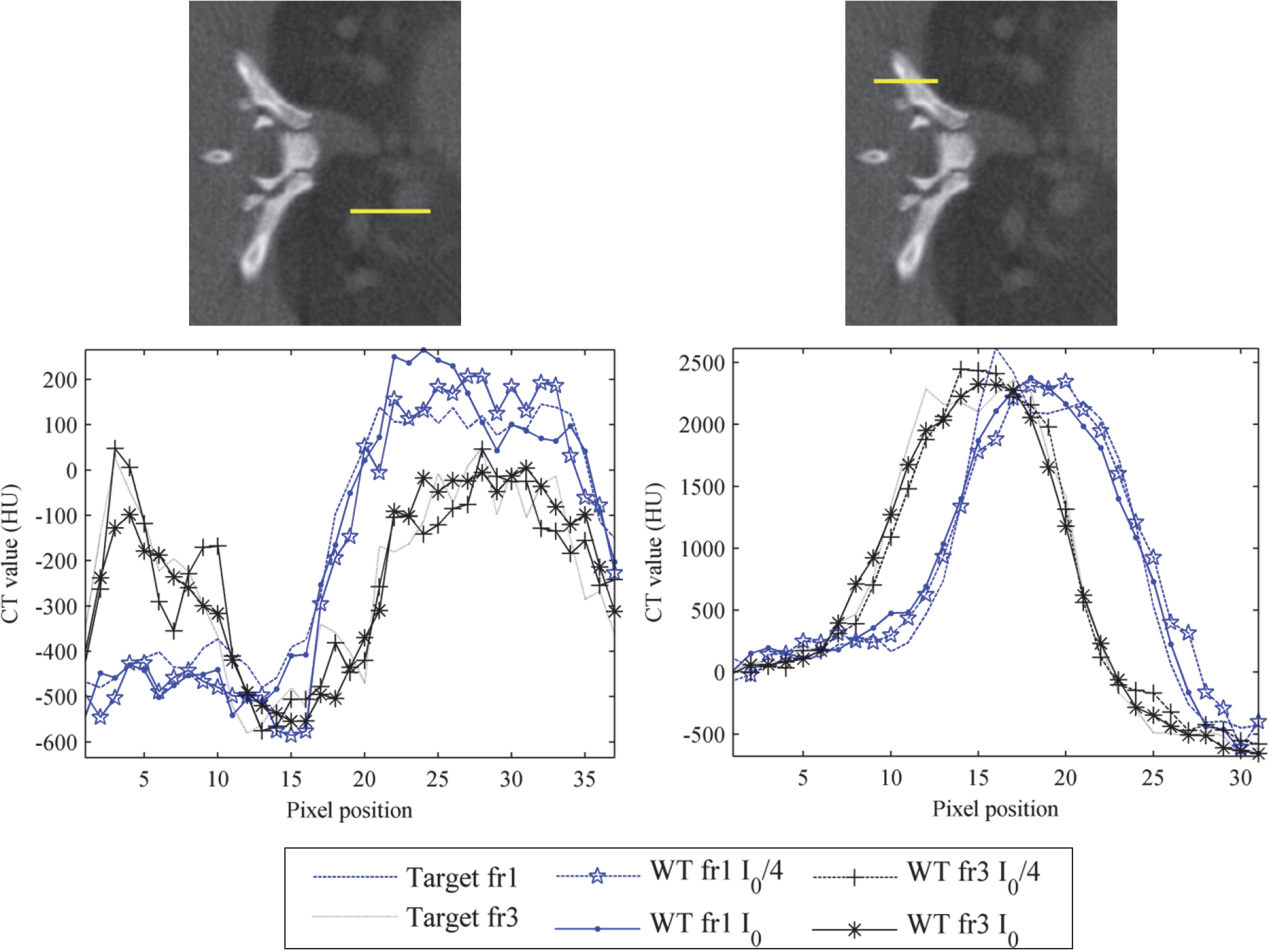
Respiratory artifact analysis. Profiles along the yellow lines in soft tissue (left) and bone tissue (right) for reference high-dose FDK (target) and for the low-dose protocol reconstructed with WT-PICCS corresponding to respiratory phases 1 and 3. The analysis shows that the reconstruction can follow the movement of the lung and vessels in the two respiratory phases.

Fig 10 shows profiles along a line containing lung tissue and vessels (Fig 10, left) and along bone tissue (Fig 10, right) for the reference high-dose FDK and WT-PICCS reconstructions of respiratory phases 1 and 3 using 120 projections and *I*_0_ = 4.5∙10^4^. The profiles reveal the existence of respiratory movement for the two respiratory phases; measuring the separation between profiles for frames 1 and 3 provides an estimate of motion of 0.5 mm. However, profiles for WT-PICCS fit the reference case well, with an error of less than 6 μm for most points in the curve.

**Fig 11:**
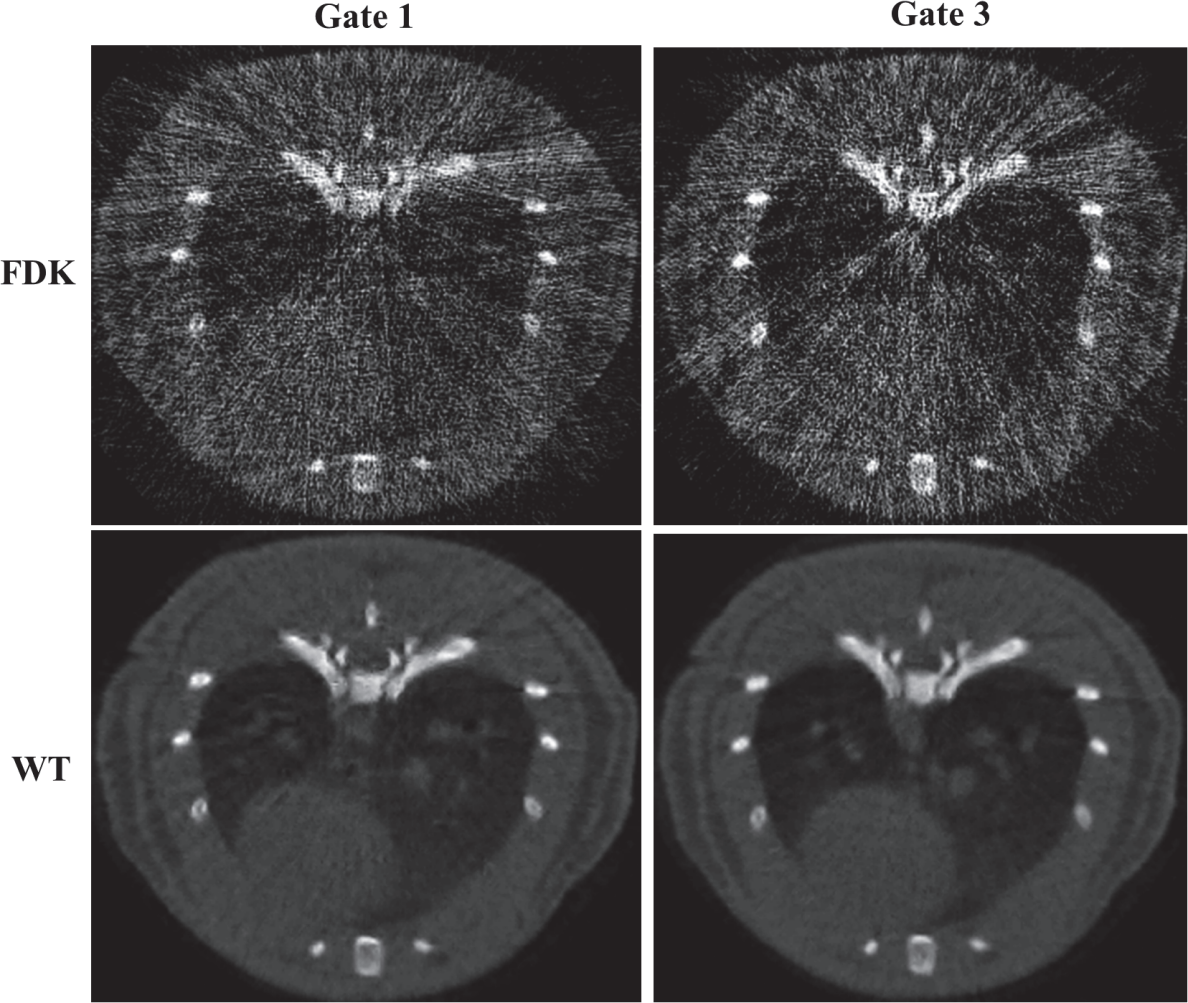
Images of respiratory phases one and three corresponding to the reference protocol for static studies reconstructed with FDK and WT-PICCS.

Fig 11 shows phases one and three, reconstructed with FDK and WT-PICCS. While FDK is highly affected by incomplete projections and noise, which hinders details and differences between phases, WT-PICCS is able to remove streak and motion artifacts, recovering the differences between phases.

## Discussion

We evaluated the suitability of different sparsity transforms (unitary, gradient, and wavelet) within the PICCS formulation for reducing dose in CT respiratory gating. Performance was assessed in different scenarios, corresponding to different X-ray flux levels and number of projections, and for different weights of the prior penalty term. Overall, our results show that the selection of the sparsity transform for the prior term does not affect spatial resolution, temporal resolution or noise performance, but has an influence on the final image texture: the wavelet transform showed a more natural pattern than the gradient and unitary transforms. While decreasing the X-ray flux leads to higher image noise, decreasing the number of projections does not have a large influence on the image noise but leads to more streak artifacts, although we did not detect significant differences between TV-PICCS and WT-PICCS. Decreasing the number of projections or the X-ray flux lowered the recovered contrast in bone and lung. While TV-PICCS and WT-PICCS presented similar profiles across soft tissue and lung, WT-PICCS led to more blurred edges for small structures than TV-PICCS probably because of the patchy-like pattern of TV regularization. Both TV-PICCS and WT-PICCS greatly outperformed FDK.

For the three reconstruction methods the results depended on the prior weight, α. For low α, all the methods converged to a solution very similar to that of the spatial TV method, which presented a patchy pattern. Increasing the prior weight reduced this pattern to some extent for all the methods. The best results for TV-PICCS and WT-PICCS were obtained using a large prior weight (α = 0.8), while for L1-PICCS the best results appeared at an intermediate weight (α = 0.5), as a large weight was prone to pepper and salt artifacts. Overall, the WT-PICCS proved to be more robust against the α value, regarding the production of artifacts. These results are consistent with prior reports on the performance of the TV-PICCS method, which found optimal α values in the range of 0.5 to 0.8, with no decrease in spatial resolution and a texture similar to that of the prior [17, 18, 20, 23, 37].

Few studies compared sparsity transforms. In this work, we compared the gradient transform, generally used for CT, with the pixel domain and the symmlet-8 wavelet transform. Other transforms such as overcomplete wavelet transform, shearlets, curvelets, and dictionary learning based sparse representation have been proposed [28, 38, 39] but were not included in our comparison.

There are some limitations in this study. Although we have evaluated the methods under several scenarios, varying the x-ray source intensity and the number of projections per phase, the effect of some variables such as image pixel size has not been assessed. In theory, smaller pixel size for lower order basis functions would result in similar results as larger pixels with higher order basis functions. Thus, the smoother results obtained by using WT or another transform instead of TV may be less relevant for smaller pixel size. However, decreasing the pixel size demands unnecessary higher computational cost and in [11] it was found to lead to poor results because the system becomes more underdetermined.

With regard to the reconstruction algorithm, we studied the influence of the sparsity parameter α and verified that varying the rest of parameters (μ, λ, and γ) within a certain range did not noticeably change the results. The most important parameter is μ,which weights the data constraint and thus affects the convergence speed. Higher values of μ speed up convergence, although they must remain sufficiently small to guarantee convergence [30]. The values are usually chosen empirically [25]. In fact, it has been shown that results for the Split Bregman method are independent of the actual value of μ, provided that it is sufficiently small and that the number of iterations is large [25]. Thus, there is no need to carefully optimize the weighting parameters, as opposed to unconstrained optimization methods, where regularization parameters have to be cautiously selected (for example with the L-curve or U-curve method [40, 41]. This is an additional advantage of the Split Bregman formulation. However, one still has to choose the number of iterations. In this work, since the high-dose data are known, we selected the iteration number that minimized the solution error taking the high-dose images as a reference, but further work is required to select a suitable number of iterations when the target image is not available.

Further improvements could be made to the proposed method. With regard to the Split Bregman formulation, in [27] the authors included statistical data modeling in a similar alternating approach, thus improving convergence. As for the prior image, although we used one prior image based on the average of data for all phase bins, other priors, such as a running average, could be used [17]. Furthermore, in this study, compressed sensing was modeled as a convex L1-norm problem. An alternative could be to propose an equivalent non-convex L0-norm problem and solve it using memetic algorithms, an improved version of evolutionary algorithms, which exploit the available information on the problem [42, 43].

In conclusion, we compared different methodologies for the reconstruction of low-dose CT data with respiratory gating based on the PICCS approach using different transforms for the prior term. Our results show that, although the gradient transform is widely used, the wavelet transform could reduce the formation of patchy cartoon-like artifacts.
